# Biogenic copper and copper oxide nanoparticles to combat multidrug-resistant *Staphylococcus aureus*: Green synthesis, mechanisms, resistance, and future perspectives

**DOI:** 10.1016/j.btre.2025.e00896

**Published:** 2025-05-06

**Authors:** Gamal M. El-Sherbiny, M.E. Shehata, Mohamed H. Kalaba

**Affiliations:** Botany and Microbiology Department, Faculty of Science, Al-Azhar University, Nasr City, Cairo, 11884, Egypt

**Keywords:** *Staphylococcus aureus*, Biogenic synthesis of copper nanoparticles (Cu-NPs), Copper oxide nanoparticles (CuO-NPs), Antibacterial properties, Mechanisms of action, Characterization techniques

## Abstract

Antimicrobial resistance has increased alarmingly in recent years, with the World Health Organization identifying multidrug-resistant *Staphylococcus aureus* as a particular threat to global public health due to its extensive resistance profile and associated high mortality rates. While various metal nanoparticles have been explored as antimicrobial agents, the specific advantages of biosynthesized copper nanoparticles against MDR *S. aureus* remain inadequately consolidated in the literature.

**Objective:**

This review uniquely evaluates the emerging evidence for biosynthesized copper nanoparticles as a sustainable, cost-effective, and potentially alternative to conventional antibiotics against multidrug-resistant *S. aureus* strains.

**Methods:**

We systematically analyzed current literature on green synthesis methods for copper and copper oxide nanoparticles, their characterization techniques, antimicrobial mechanisms, and efficacy against multidrug-resistant *S. aureus*, focusing on identifying knowledge gaps and future research directions.

**Results:**

Unlike other metal nanoparticles, biosynthesized copper nanoparticles demonstrate significant antibacterial activity against multidrug-resistant *S. aureus* through multiple simultaneous mechanisms that bacteria try to develop resistance against. Their unique physicochemical properties enable enhanced bacterial elimination compared to conventional antibiotics and other metal nanoparticles, with minimal toxicity to mammalian cells at therapeutic concentrations. Our analysis further reveals the considerable potential of these nanoparticles to overcome existing biological barriers in infection sites that limit conventional therapies.

**Conclusion:**

This broad assessment of biosynthesized copper nanoparticles shows strong potential as a therapy against MDR *S. aureus* and provides a foundation for future research to address antimicrobial resistance where current treatments fail.

## Highlights

• Multidrug-resistant *Staphylococcus aureus* represents a worldwide health crisis, contributing to rising rates of illness and death.
• Techniques applied in the fabrication of Cu/CuO nanoparticles.
• Antibacterial activity of Cu/CuO nanoparticles against Multidrug-resistant *Staphylococcus aureus*.
• Methods characterization of biosynthesis of Cu/CuO nanoparticles.

## 1 Introduction

*Staphylococcus aureus* is a Gram-positive coccal bacterium that typically goes unnoticed as a component of our normal microbiota; however, it can occasionally present a significant risk to human health through persistent infections. This bacterium is responsible for a diverse array of diseases, which include skin and soft tissue infections such as impetigo, folliculitis, carbuncles, furuncles, cellulitis, and scalded skin syndrome, as well as more severe conditions like infective endocarditis, bacteremia, osteomyelitis, septic arthritis, pulmonary infections (including pneumonia and empyema), infections associated with prosthetic devices, gastroenteritis, urinary tract infections, toxic shock syndrome, and meningitis [1]. The multidrug resistance exhibited by this bacterium renders it one of the most challenging pathogenic organisms to treat in the annals of antibiotic therapy, compounded by its ability to evade the human immune response. Since the 1940s, nearly every antibiotic developed has been rendered ineffective against it. The initial identification of methicillin-resistant *Staphylococcus aureus* (MRSA) occurred in clinical samples of *S. aureus* in 1961. Following this discovery, MRSA evolved into a multi-resistant pathogen prevalent in healthcare settings and has since been disseminated globally. In 1997, a modified susceptibility to vancomycin was noted in the Mu50 MRSA strain, which represented an adaptive mutation of *S. aureus* against vancomycin, a drug that has historically served as the last line of defense against MRSA infections, leading to the classification of vancomycin-intermediate *S. aureus* (VISA) according to CLSI standards [2]. Methicillin-resistant *Staphylococcus aureus* (MRSA) strains that demonstrate resistance to β-lactam antibiotics are associated with the presence of transferable genomic islands, referred to as SCCmec (staphylococcal chromosomal cassette mec). Within these islands, the mec gene is responsible for conferring methicillin resistance. These genomic islands are characterized by a high abundance of mobile genetic elements and are subject to rapid evolutionary changes. Various forms of SCCmec may harbor the mecA or mecC genes, along with resistance genes targeting other antibiotic classes, including aminoglycosides, lincosamides, macrolides, streptogramins B, and tetracyclines [3]. *Staphylococcus aureus* has been classified as part of the multidrug-resistant ESKAPE group, which comprises the most critical pathogenic bacteria, including *Enterococcus faecium, Staphylococcus aureus, Klebsiella pneumoniae, Acinetobacter baumannii, Pseudomonas aeruginosa*, and *Enterobacter* species [4]. *S. aureus* holds a unique status due to its relatively high virulence, coupled with significant plasticity that allows it to adjust to diverse environmental conditions [5]. Treatment options for infections resulting from multidrug-resistant bacteria (MDRB) are frequently limited when it comes to antibiotic therapies. In this context, multidrug-resistant *Staphylococcus aureus* (MRSA) is responsible for a significant number of lethal infections within hospital environments [6]. The aforementioned medical issues highlight the urgent necessity for the development of novel strategies aimed at eradicating multidrug-resistant bacteria (MDRB). Given the stagnation in antibiotic development and the increasing prevalence of bacterial resistance, treatment modalities that utilize nano-therapy are critically important. In this context, nanoparticles have the potential to address these challenges and enhance the efficacy of therapeutic agents [2,7].

Researchers have created advanced technologies aimed at reducing the risk of infections by enabling pathogens to interact with specially formulated treatments or specific carriers known as nanoparticles [8]. In this context, nanotechnology has emerged as a particularly promising field in the fight against multidrug-resistant bacterial infections. The dimensions of nanomaterials typically range from 1 to 100 nanometers (nm). Nano-antibiotics are increasingly recognized in the realm of nanomedicine due to their potential to transform the treatment of antibiotic-resistant bacteria [9]. To enhance the efficacy, precision, and delivery of antibiotics, a variety of nanoparticles are employed. These include membrane-bound nanoparticles, metallic nanoparticles, metal oxides, carbon-based nanoparticles, chitosan nanoparticles, mesoporous nanoparticles, as well as solid lipid nanoparticles, nanocomposites, nanosheets, and nano-mesh structures [10].

Several methodological approaches exist for synthesizing copper and copper oxide nanoparticles, each with distinct advantages for antimicrobial applications. Traditional physical and chemical methods include chemical reduction, thermal decomposition, electrochemical synthesis, and sol-gel techniques. However, our focus lies on green synthesis methods, which employ biological materials as reducing and stabilizing agents. These methodologies utilize plant extracts, microbial cultures, or biomolecules to facilitate the controlled formation of copper nanoparticles. Green synthesis offers significant advantages through reduced environmental impact, enhanced biocompatibility, and cost-effectiveness compared to conventional methods [11]. The biological synthesis of copper nanoparticles typically follows a three-phase process: (1) an activation/reduction phase where copper ions are reduced to copper atoms; (2) a growth phase where small copper clusters form; and (3) a termination phase where bioactive molecules stabilize the nanoparticles, preventing aggregation [12,13]. Currently, inorganic nanoparticle production is dominated by chemical and physical methodologies despite their numerous disadvantages, including high investment requirements, potential toxicity, inefficient yields, and environmental concerns. As a result, a shift toward biological synthesis approaches for nanoparticle creation is gaining momentum [14]. Awwad et al. [15] indicated that both Gram-positive and Gram-negative pathogenic bacterial strains are vulnerable to bio-fabricated copper nanoparticles. Additionally, the biogenically synthesized copper and copper oxide nanoparticles demonstrated antibacterial properties against *Staphylococcus aureus, Pseudomonas aeruginosa, Escherichia coli,* and *Clostridium difficile* [2,15]. Copper nanoparticles (Cu-NPs) synthesized using *Gloriosa superba* leaf extract showed antimicrobial activity against both the gram-negative *Klebsiella aerogenes* and gram-positive *Staphylococcus aureus* [16]. Green-synthesized CuO-NPs using *Sida acuta* leaf extracts show considerable potential as antimicrobial agents in textile applications, with research confirming that *Sida acuta*-coated CuO-NPs can inhibit both Gram-negative and Gram-positive bacterial growth on cotton fabric surfaces [17]. Copper nanoparticles bio-fabricated using water-soluble polysaccharides (SP) extracted from the brown seaweed *Sargassum vulgare* exhibited notable antibacterial and antibiofilm activities against methicillin-resistant *Staphylococcus aureus* (MRSA) [14].

The characterization and detection of synthesized copper nanoparticles employ multiple complementary techniques to confirm their formation and structure. UV–visible spectroscopy serves as a primary technique for confirming nanoparticle formation through surface plasmon resonance bands, which vary depending on the size of copper nanoparticles. X-ray diffraction (XRD) analysis provides crystallographic information, confirming the crystalline nature and phase purity of the nanoparticles. Fourier Transform Infrared Spectroscopy (FTIR) identifies the functional groups involved in nanoparticle formation and stabilization while Scanning Electron Microscopy (SEM), Transmission Electron Microscopy (TEM), and Atomic Force Microscopy (AFM) provide detailed morphological and dimensional analysis [[18], [19], [20]].

Copper nanoparticles represent a promising solution due to several critical advantages: (1) their multi-target mechanisms of action, which reduce the likelihood of resistance development [21]; (2) enhanced penetration into bacterial biofilms, a significant challenge in treating persistent infections [22]; (3) potential synergistic effects when combined with conventional antibiotics [23] and (4) relatively low toxicity to human cells when properly engineered [24]. Additionally, the green synthesis methods explored in this study align with sustainable development goals, offering environmentally friendly alternatives to conventional nanoparticle synthesis approaches.

This study addresses a significant gap in the literature by systematically evaluating the efficacy of biogenically synthesized copper nanoparticles against clinical isolates of multidrug-resistant *S. aureus*, providing valuable insights for developing novel therapeutic strategies in an era of diminishing antibiotic efficacy.

## 2 Biosynthesis mechanism of Cu/CuO-NPs

Various oxidation states of copper ions include (I) Cu, (II) Cu, and a limited quantity of (III) Cu ions. In the process of synthesizing copper and copper oxide nanoparticles using extracts from bacteria, actinomycetes, fungi, algae, and plants, the formation of CuO, Cu_2_O, and Cu_4_O_3_ occurs at a consistent rate, irrespective of the concentration, pH, and temperature of the precursor. Nevertheless, these parameters significantly affect the type of copper particles produced during the green synthesis process. During this process, the biomolecules present in the extract facilitate the oxidation and reduction of Cu^2+^ ions to the Cu^0^ state, leading to the formation of copper oxide nanoparticles. Additionally, certain biomolecules in the extract function as capping agents, contributing to the stabilization of the nanoparticles formed [2].

## 3 Synthesis methods of Cu/CuO-NPs

The method employed to produce materials from environmentally friendly or sustainable sources, utilizing an effective reducing agent, solvent, and non-toxic additives, is referred to as green synthesis. Copper nanoparticle biosynthesis is recognized as a "green methodology" due to its several quantifiable advantages over conventional methods. This approach utilizes renewable biological resources like plant extracts and microorganisms as reducing and capping agents, eliminating the need for toxic chemicals [25]. Biosynthesis is more energy-efficient than physical methods, as it operates under mild conditions, typically at ambient temperature. This contrasts with high-temperature processes (>300 °C) used in conventional methods. Additionally, biosynthesis generates significantly less hazardous waste compared to chemical reduction methods, thanks to the use of non-toxic biological agents and eco-friendly solvents [26]. Additionally, Life cycle assessments (LCAs) further support the environmental benefits of biosynthesis. These assessments indicate that biosynthesis significantly reduces environmental impact factors such as carbon footprint, resource depletion, and ecotoxicity potential [13].

The biosynthesis process also involves the use of substances to ensure stability [27]. This synthesis method produces more stable molecules and is characterized by its simplicity, cost-effectiveness, predictability, sustainability, and a degree of reproducibility. Consequently, researchers are keen to employ this biosynthetic approach to develop various nanomaterials, including hybrid materials, bioinspired materials, and metal/metal oxide nanoparticles. Therefore, green synthesis is widely recognized as a crucial technique for mitigating the negative impacts associated with conventional nanoparticle synthesis methods used in both laboratory and industrial settings [28]. Traditional approaches to nanoparticle fabrication, including chemical and physical synthesis, are costly, hazardous, and detrimental to the environment. Additionally, the chemical synthesis processes involved in nanoparticle production can sometimes influence biological activities due to various factors, including size distribution, shape, surface charge, surface chemistry, and the presence of capping agents [29].

When comparing the quality of nanoparticles produced through different methods, several studies have demonstrated that biogenic synthesis can yield particles with enhanced properties for antimicrobial applications. Siddiqi and Husen [30] found that plant-derived copper nanoparticles exhibited superior stability in physiological conditions compared to chemically synthesized counterparts, with aggregation rates reduced by approximately 40–60 % over 72 hours. This enhanced stability directly correlates with improved efficacy against multidrug-resistant pathogens, as demonstrated by Hall et al. [31], who reported that glutamic acid-coated copper oxide nanoparticles (GA-CuONPs) maintained antimicrobial activity against multidrug-resistant pathogens for extended periods, significantly outperforming conventional particles. Specifically, GA-CuONPs adhered to medical-grade materials and exhibited stable antimicrobial action over prolonged durations, effectively reducing biofilm biomass and viable bacterial cells even in physiologically relevant conditions, such as plasma-like media. These comparative advantages are particularly relevant for potential clinical applications against persistent MRSA infections, where sustained antimicrobial activity is essential for therapeutic success.

**Fig. 1:**
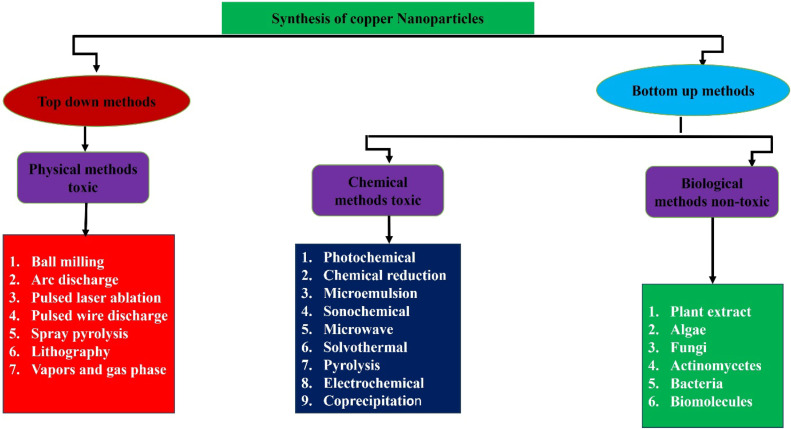
Synthesis methods of copper and copper oxide nanoparticles. Classification of synthesis methods into Top-Down (physical, toxic) and Bottom-Up (chemical, toxic; biological, non-toxic) approaches. Physical methods involve breaking bulk materials into nanoparticles, while chemical and biological methods build nanoparticles from molecular precursors. Biological methods offer a green alternative using plant extracts, algae, fungi, and bacteria.

Researchers have pinpointed specific green pathways, which refer to naturally occurring substances and their resultant products, that can be utilized to synthesize nanoparticles to mitigate adverse effects. There are two distinct methodologies for nanoparticle synthesis, as illustrated in the accompanying Fig. (1): (i) the top-down approach focuses on the physical processes involved in the synthesis of nanoparticles, and (ii) the bottom-up method prioritizes chemical and biological techniques.

Recent comparative studies between top-down and bottom-up approaches have revealed significant differences in nanoparticle properties relevant to antimicrobial applications. Previous analysis demonstrated that bottom-up biosynthetic approaches, such as those using plant extracts or microbial agents, produce copper nanoparticles with more uniform size distributions, reflected in lower polydispersity indices (0.15–0.25) compared to top-down methods (0.3–0.5). These nanoparticles also exhibit higher surface reactivity due to their controlled synthesis and reduced aggregation. This enhanced surface activity directly correlates with improved antibacterial efficacy, particularly against MRSA strains, as the uniform size and increased surface area facilitate better interaction with bacterial cells and biofilms [26,32]. The relationship between synthesis methodology and antimicrobial efficacy was further elucidated by previous reports, which support the statement that biogenically synthesized copper oxide nanoparticles (CuO NPs) exhibit superior bioactivity per unit mass compared to those produced through physical methods. Specifically, biogenic CuO NPs synthesized using fungal or bacterial cultures demonstrated comparable antimicrobial activity at concentrations 30–40 % lower than chemically or physically synthesized counterparts. This enhanced efficacy is attributed to their smaller size, higher surface-to-volume ratio, and the presence of biologically active capping agents, which improve interaction with bacterial cells and reduce aggregation rates in physiological conditions [22,33,34]. Fig. (1): illustrates the distinct methods for the green production of nanoparticles.

Phyto methods, such as using excerpts of plants and plant resources.

Microbial methods include the use of microorganisms such as algae, actinomycetes, fungi, and bacteria.

Bio-template approaches, such as using viruses, diatoms, and membranes as templates.

When comparing these three green approaches, notable differences in scalability and reproducibility have been documented in recent literature. Phyto-based methods generally demonstrate the highest reproducibility with batch-to-batch variation in particle size typically under 10 %. This is attributed to the uniformity of bioactive compounds in plant extracts, which act as stabilizing and reducing agents, ensuring controlled nucleation and growth processes during synthesis as reported by Antonio-Pérez et al**.** [26]**.** In contrast, microbial synthesis methods show wider variability (15–25 %) but offer superior yields for large-scale production, with bacterial systems capable of producing up to 2.5 times the nanoparticle mass per unit input compared to plant-based systems according to comparative studies by Singh et al. [35]. Bio-template approaches, while offering precise morphological control, present significant challenges for large-scale implementation, limiting their current application primarily to specialized high-value applications rather than bulk antimicrobial production [36].

To create nanoparticles, both extracellular and intracellular biological techniques have been used. Although the specific process for producing nanoparticles using biological agents has not yet been found, it is hypothesized that specific biomolecules are involved. Additionally, the processes for forming intracellular and extracellular nanoparticles are different, and it seems that extracellular enzymes are important for the synthesis of extracellular nanoparticles whereas the cell wall of microorganisms is important for the synthesis of intracellular nanoparticles. Due to its easier synthesis process and higher production rate, extracellular nanoparticle synthesis has become more popular than intracellular nanoparticle synthesis [37]. Comparative analyses support this preference, demonstrating that extracellular approaches achieve significantly higher production rates, often 3–4 times greater than intracellular methods, due to the simplicity of harvesting nanoparticles directly from the reaction medium without the need for complex cell lysis or extraction processes. Additionally, extracellularly synthesized copper nanoparticles exhibit superior stability in biological media, attributed to natural capping by microbial proteins and polysaccharides, which enhances their resistance to aggregation and ensures sustained antimicrobial efficacy in complex infectious environments [38,39].

## 4 Biogenic synthesis of Cu/CuO-NPs

The process referred to as "green synthesis" involves the biosynthesis of nanoparticles utilizing various organisms, including plants, algae, bacteria, fungi, and actinomycetes. This approach aligns with the principles of "green chemistry," aiming to produce clean and environmentally benign nanoparticles. The utilization of these microorganisms and plants offers a sustainable method for generating nanoparticles with distinctive properties. In these biosynthetic processes, both unicellular and multicellular organisms participate in the reactions. Plants, often regarded as nature's chemical factories, are particularly valued for their low maintenance requirements and cost-effectiveness in cultivation. Furthermore, plants exhibit a remarkable capacity for the accumulation and detoxification of heavy metals, which is crucial in mitigating environmental pollutants, especially since even trace amounts of these metals can pose significant risks [14,40].

a. **Biosynthesis of Cu/CuO—NPs using plant extracts**

**Fig. 2:**
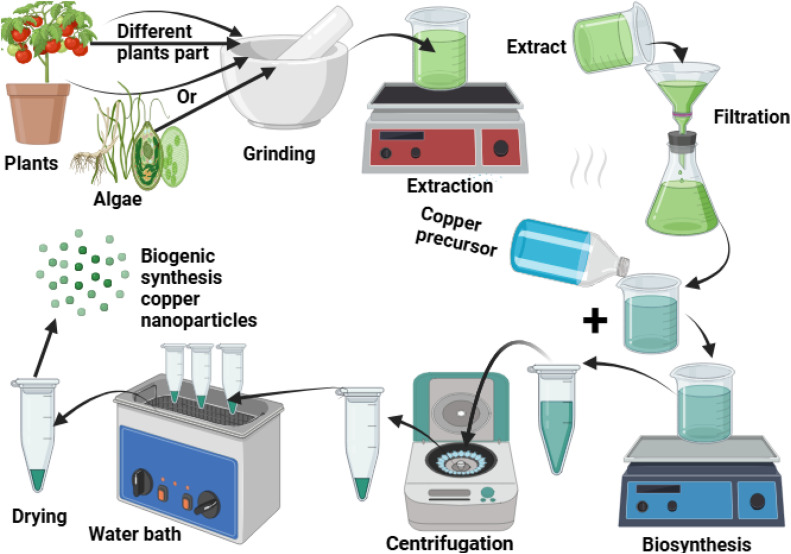
Biogenic synthesis of copper nanoparticles using different plant parts and algae. Schematic of green synthesis using plant/algae extracts. Extracted bioactive compounds react with a copper precursor for nanoparticle formation, followed by centrifugation and drying to obtain biogenic copper nanoparticles.

The application of plant extracts for the synthesis of nanoparticles offers several benefits compared to alternative biological synthesis methods, including those that utilize microorganisms. Notably, the synthesis of metal nanoparticles through plant extracts is remarkably prevalent [41], much faster, and remarkably mono-dispersive, compared to other biological techniques [42]. The main challenges associated with the utilization of microorganisms involve the complex incubation procedures, the harmful nature of certain bacteria, and the difficulties in isolating microorganisms, which render them unsuitable for many researchers. Consequently, plant extracts serve as a highly effective alternative for the synthesis of metal and metal oxide nanoparticles [11,43]. In addition, the reaction kinetics of plant-assisted nanoparticle synthesis is significantly more rapid when compared to other biosynthetic methods that are analogous to chemical nanoparticle synthesis. Various plant materials, including fruits, leaves, stems, and roots, have been extensively utilized in this environmentally friendly approach to nanoparticle production due to the remarkable phytochemicals they contain as detailed in Table 1. Consequently, copper oxide nanoparticles have been widely produced using a range of plant extracts for the reasons outlined above [44]. In this plant-based manufacturing process, metal salts are integrated with plant extracts, and the reaction is completed within 1 to 3 hours at ambient temperature (Fig. 2). Recent innovations in plant-mediated synthesis have significantly expanded the range of effective botanical sources for copper nanoparticle production. Notably, Kausar et al. [19]. demonstrated that *Annona muricata* (soursop) fruit extract serves as an exceptional reducing and capping agent for CuO nanoparticle synthesis. Their method produced well-defined nanoparticles with average diameters of 35–54 nm that exhibited dual functionality: potent antimicrobial activity against pathogenic bacteria and selective cytotoxicity against MCF-7 breast cancer cell lines through ROS-mediated apoptosis induction. This bifunctional therapeutic capability represents a significant advancement over conventional single-function copper nanoparticle systems and indicates the expanding potential of biogenically synthesized CuO—NPs for integrated antimicrobial and anticancer applications. In parallel developments, Mohanaparameswari et al. [45], reported similar multifunctional properties with AgO nanoparticles synthesized using *Solanum nigrum* and Mentha leaf extracts, although comparative stability studies indicate that copper-based nanoparticles maintain superior structural integrity in physiological environments due to their distinctive oxidation characteristics and surface stabilization by plant-derived polyphenolic compounds. Various bioactive compounds present in the plant extracts, including proteins, terpenoids, alkaloids, tannins, flavonoids, and phenols, function as reducing agents and stabilizers, facilitating the transformation of metallic ions into nanoparticles. The electrons generated by the plant extract serve to reduce copper salts. As the phytochemicals interact with copper ions, a reduction occurs, resulting in the formation of copper oxide nanoparticles [14,46].

a. **Biosynthesis of Cu/CuO—NPs by algae**

**Table 1:** Antibacterial activity of plant biogenic Cu/CuO—NPs against MRSA, MSSA, and some bacterial species.

**Plants / precursor**	**Part used**	**Size (nm)**	**Shape**	**Concentration**	**Test organisms**	**Ref.**
**Copper acetate**						
*Averrhoa carambola*	Fruit	15	Spherical, hexagonal	20 µg/mL	*S. aureus, Bacillus* spp., *Pseudomonas* spp.	[99]
*Ocimum tenuiflorum*	Leaves	12–44	Spherical	3125–12,50 µg/mL	*S. aureus, B. subtilis, E. coli*	[100]
*Aloe-vera*	Leaves	45–95	Elliptical	1562 µg/mL	*S. aureus, B. subtilis, E. coli*	[101]
*Camellia Sinensis*	Leaves	25–32	Crystalline	1000 µg/mL	*S. aureus, B. subtilis, E. coli, K. pneumonia*	[102]
*Polyalthia longifolia*	Leaves	50–60	Quasi-spherical	100–1000 µg/mL	*S. aureus, S. pyogenes, E. coli, P. aeruginosa*	[103]
*Clematis orientalis*	Leaves	13–53	Crystalline	0.25 M	*S. aureus, B. subtilis, E. coli, P. aeruginosa, K. pneumoniae*	[104]
**Copper nitrate**						
*Allium sativum*	Root	20–40	Circular	150 µg/mL	*S. aureus, E. coli. B. subtilis, P. aeruginosa, K. pneumoniae*	[105]
Ginger and garlic	Root	20–45	Spherical, agglomerated	1000 µg/mL	*MDR S. aureus*	[106]
*Tinospora cordifolia*	Leaves	10	Spherical	1001 µg/mL	*S. aureus, K. aerogenes, E. coli, P. desmolyticum*,	[107]
*Cordia sebestena*	Flower	20–35	Spherical	75 µg/mL	*S. aureus, B. subtilis, E. coli, K. pneumoniae*	[108]
*Gloriosa superba* L.	Leaves	5–10	Spherical	1000 µg/mL	*S. aureus, K. aerogenes, P. desmolyticum, E. coli*	[109]
*Eryngium caucasicum*	Leaves	40	Spherical	30–100 µg/mL	*S. aureus, E. coli, S. typhimurium, B. cereus*	[110]
*Saccharum officinarum*	Stem	5–140	Spherical	100 µg/mL	*S. aureus, E. coli, P. aeruginosa, B. subtilis*	[111]
*Madhuca longifolia*	Flower/seeds	30–100	Spherical	10 mg/mL	*S. aureus, E. coli, B. subtilis*	[112]
				4 mg/mL	*S. aureus, P. aeruginosa*	
				8 mg/mL	*S. aureus, P. aeruginosa*	
*Citrus aurantifolia*	Leaves	10	Spherical, crystalline	50 µg/mL	*S. aureus, K. pneumoniae*	[113]
*Illicium verum*	Fruit	150–220	Undefined	100 µg/mL	*S. aureus*	
*Myristica fragrans*	Fruit	210–270	Undefined	100 µg/mL	*S. aureus*	
*Ruellia tuberosa*	Leaves	82	Spherical	75 µg/mL	*S. aureus, K. pneumoniae, E. coli*	[114]
*Solanum lycopersicum*	Leaves	20–40	Spherical	300 µg/mL	*S. aureus, B. subtilis, E. coli*	[115]
*Allium saralicum*	Leaves	45–50	Spherical	8 mg/mL	*S. aureus, P. aeruginosa, E. coli, B. subtilis, S. typhimurium*,	[116]
*Achillea Nobilis*	Flower	15–25	Hexagonal	50 µg/mL	*S. aureus, E. coli*	[117]
*Achyranthes aspera*	Leaves	95	Spherical	250 mM	*S. aureus, P. aeruginosa*	[118]
*Citrus aurantifolia*	Leaves	22	Crystalline	150 µg/mL	*S. aureus, E. coli*	[119]
*Mentha pulegium*	Leaves	21–48	Spherical	1000 µg/mL	*S. aureus, B. cereus, E. coli, K. pneumoniae*	[120]
*Brassica oleracea* var.	Leaves	77.5	Spherical	50 µg/mL	*S. aureus, E. coli*	[121]
*Thymbra spicata*	Leaves	21–26.8	Spherical	100 µg/mL	*S. aureus, B. cereus, E. coli, S. typhimurium*	[122]
*Prunus mahaleb* L.	Whole	10–50	Spherical	0.5–2 mg/mL	*S. aureus, K. pneumonia, P. aeruginosa, E. coli*	[123]
**Copper chloride**						
*Ficus carica*	Leaves	41.5	Spherical	200 µg/mL	*S. aureus, Candida* spp., *Aspergillus* spp., *A. baumanii*	[124]
*Tinospora cardifolia*	Leaves	63–143	Spherical	175 µg/mL	*S. aureus, E. coli*	[125]
*Vitex negundo*	Root	40–60	Spherical, hexagonal	500 µg/mL	*S. aureus, B. subtilis, E. coli, P. aeruginosa*	[126]
*Cardiospermum halicacabum*	Leaves	40	Hexagonal	50 µg/mL	*S. aureus, P. aeruginosa, E. coli*	[127]
*Brassica oleracea, Solanum tuberosum, Pisum sativum*	Peels	32.5, 40.75, 47.2	Spherical and cubical	45 µg/mL	*S. aureus, P. aeruginosa, E. coli, B. subtills*	[128]
**Copper sulphate**						
*Morinda citrifolia*	Whole	10–30	Spherical	————-	MRSA (ATCC 4330), *S. aureus* (ATCC 33,591), *S. epidermidis* (ATCC 35,984*), Salmonella typhi, E. coli* (ATCC 25,922)	[129]
*Cymbopogon citratus*	Leaves	2–22	Spherical, hexagonal	500–2000 µg/mL	MRSA*, S. aureus*	[130]

Algal species are regarded as significant microorganisms in the biosynthesis of CU/CuO—NPs, particularly as nanoparticle sizes between 5 and 45 nm and 6 to 7.8 nm have been successfully generated utilizing a boiling aqueous extract from the brown algae *Cystoseira trinodis* [47], and *Bifurcaria bifurcata* [48] as detailed in Tables 2. Ramaswamy et al. [49] reported the utilization of an aqueous extract derived from *Sargassum polycystum* for the synthesis of CuO—NPs. Additionally, the aqueous extract of *Botryococcus braunii* was found to produce CuO—NPs with dimensions ranging from 2 to 10 nm [50]. Furthermore, Bhattacharya et al. [51] adopted a slightly modified approach by heating the extract to 50 °C instead of boiling it, which resulted in an aqueous extract from *Anabaena cylindrica* capable of synthesizing CuO—NPs with a particle size of 3.6 nm. Consequently, various algal species demonstrated reduction and stabilization processes that employed copper as a promoter, alongside their diverse organic compounds.

a. **Biosynthesis of Cu/CuO—NPs using bacteria**

**Table 2:** Antibacterial activity of algae biogenic Cu/CuO—NPs against MRSA, MSSA, and some bacterial species.

**Algae/precursor**	**Part used**	**Size (nm)**	**Shape**	**Concentration**	**Test organisms**	**Ref.**
**Copper nitrate**						
*Cystoseira trinodis*	Biomass	6–7.8	Crystalline	10 µg/mL	*S. aureus, E. coli, E. faecalis, S. typhimurium, B. subtilis*,	[131]
**Copper sulphate**						
*Sargassum vulgare*	Soluble polysaccharides	198	Spherical	150–250 μg/ml	*S. aureus* MRSA and MSSA	[132]
*Bifurcaria bifurcata*	extract	18.34	Spherical	20 μl	*S. aureus, E. aerogenes*	[133]
*Cystoseira trinodis*	Extracts	6 - 7.8	Crystallite	2.5–10.0 μg/ml	*S. aureus E. coli, E. faecalis, S. typhimurium, B. subtilis*,	[132]
**Copper acetate**						
*Botryococcus Braunii*	Extracts	10–70	———	—–	*S. aureus* MTCC96, *P. aeruginosa* MTCC 441, *E. coli* MTCC442, *K. pneumoniae* MTCC 109	[134]

**Fig. 3:**
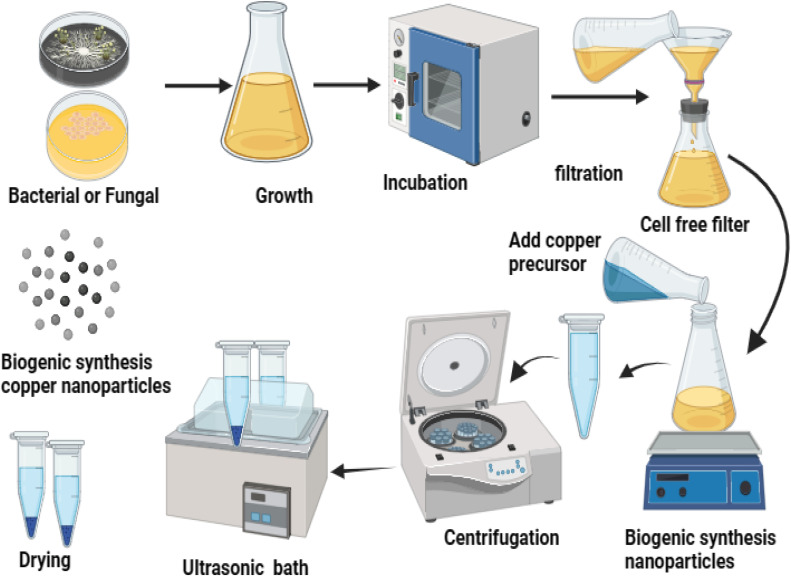
Biogenic synthesis of copper nanoparticles using fungi and bacteria species. Bacterial/fungal cultures are grown, incubated, and filtered to obtain a cell-free extract. The addition of a copper precursor enables nanoparticle biosynthesis, followed by centrifugation, ultrasonic treatment, and drying to obtain biogenic copper nanoparticles.

Various bacterial species have been employed in the past year to synthesize a range of nanoparticles, including copper oxide nanoparticles [52], as detailed in Table 3. A range of materials exhibiting intriguing shapes and nanoscale dimensions has been produced through the utilization of bacteria, employing both extracellular and intracellular methods as shown in Fig. (3). The capacity of bacteria to synthesize nanoparticles is notably high. They offer numerous advantages, such as rapid production times, straightforward culture methods, safe experimental environments, enhanced stability, the formation of extracellular nanoparticles, and the ease of implementing genetic modifications [14]. It is widely recognized that bacteria adapt to environments containing toxic metals by transforming these harmful metal ions into less toxic forms, such as metal sulfides or oxides. Furthermore, it is established that bacteria can thrive in settings with elevated levels of hazardous metals by converting these toxic ions into non-toxic metal oxides [53]. Research has shown that bacteria generate a variety of significant thiol-containing compounds in response to oxidative stress. These compounds act as capping agents, preventing the oxidation of metal oxide nanoparticles during the bacterial synthesis process [54]. Currently, the mechanisms driving the nanoscale transformations remain incompletely understood. Additionally, moderate experimental conditions, including pH, temperature, simple post-processing, and a brief formation duration, are essential for the successful production of nanoparticles [14].

a. **Biosynthesis of Cu/CuO**—**NPs by using fungi**

**Table 3:** Antibacterial activity of bacteria biogenic Cu/CuO—NPs against MRSA, MSSA, and some bacterial species.

**Bacteria and actinomycetes/precursor**	**Part used**	**Size (nm)**	**Shape**	**Concentration**	**Test organisms**	**Ref.**
**Copper sulphate**						
*Streptomyces rochei*	Filtrate	10.7	Spherical	6.5 µg/ ml	MDR *Staphylococcus aureus, (MRSA)*	[2]
*Bacillus subtilis* (MTCC 441)	Filtrate	11.47 ± 2.6	Spherical	≤ 0.625 mg/ml	*Staphylococcus aureus MRSA, E. coli* Bi2A, *Pseudomonas aeruginosa* VTCCBAA2, *Enterobacter cloacae* Bu59.	[135]
Actinomycetes VITBN4,	Filtrate	29.82	Spherical	5–100 μg/ml	*S. aureus, B. cereus, Proteus mirabilis, Aeromonas Edwardsiella tarda, caviae, Aeromonas hydrophila Vibrio anguillarum)*	[136]
*Enterococcus faecalis*	Filtrate	20–90	————	———	*Staphylococcus aureus* MRSA, MDR *E. coli, Klebsiella pneumoniae,*	[137]

Numerous fungal species have been employed in recent years to produce copper oxide and various metal nanoparticles [52], as detailed in Table 2. Fungi exhibit a significant potential for the biosynthesis of nanoparticles when compared to other microorganisms. They demonstrate greater tolerance to agitation, shear stress, and a range of other conditions within bioreactors or alternative growth environments than bacteria. Conversely, due to the composition of their cell walls, fungi may exhibit a lower susceptibility to copper nanoparticles than their bacterial counterparts. The cell walls of fungi are primarily composed of polysaccharides, such as chitin (N-acetylglucosamine), and lipids, which confer structural integrity and protection against nanoparticles [55]. The *Trichoderm*a genus is known for producing a variety of bioactive compounds, including polyketides, terpenes, glycolipids, diketopiperazines, and numerous reductive enzymes. These enzymes facilitate the synthesis of silver and zinc oxide nanoparticles, in addition to copper oxide nanoparticles [56]. Fungi are capable of generating various types of nanoparticles through both external and internal mechanisms. Certain fungal species can produce nanoparticles that are smaller, more dispersive, and possess superior dimensions compared to those formed via the extracellular route [57]. The extracellular synthesis of nanoparticles offers several advantages, including the potential absence of cellular components in the resulting nanoparticles. This is largely because fungi synthesize numerous compounds that act as stabilizing and reducing agents, making the extracellular pathway the most commonly employed method for nanoparticle synthesis [57]. A variety of fungal strains have been utilized to produce metal oxide nanoparticles, particularly copper oxide nanoparticles. Consequently, numerous fungal species were examined for this study, revealing that fungi are excellent candidates due to their high enzyme production and ease of handling in laboratory settings. The extracellular synthesis of copper nanoparticles has been documented in species such as *Penicillium aurantiogriseum, Penicillium citrinum*, and *Penicillium waksmanii* [58]. In a notable study, Majumder [59] described a process to facilitate copper nanoparticle (Cu-NP) production at the nanoscale by cultivating *Fusarium oxysporum* at ambient temperature, followed by assessing its capability to extract copper from integrated circuits. In another significant advancement, researchers utilized the decomposing biomass of *Hypocrea lixii* sourced from a metal mine to synthesize spherical Cu-NPs with an average diameter of 24.5 nm. Subsequent infrared spectroscopy analysis revealed that amide groups in proteins contributed significantly to the stabilization and capping of these Cu-NPs [14].

## 5 **Factors affecting the biosynthesis of** Cu/CuO—NPs

The biosynthesis of Cu/CuO—NPs is influenced by a range of chemical and physical parameters. These parameters include pH, temperature, reaction time, substrate concentration, and the use of reductants. Optimizing nanoparticle synthesis parameters is a key aspect of advancing nanomaterial development, especially for antimicrobial applications [60]. Firstly, it is important to note that the extraction conditions and biological material preparation significantly influence not only the physicochemical properties of the resulting nanoparticles but also their biological compatibility and activity profiles. Comparative studies between copper and zinc oxide nanoparticles have revealed process-dependent differences in biocompatibility. Neamah et al. [61], demonstrated that ZnO nanoparticles biosynthesized using *Capparis spinosa* fruit extract exhibit excellent biocompatibility with human cells while maintaining strong antimicrobial activity, a balance that depends critically on extraction temperature and time**.** The role of pH in nanoparticle synthesis has garnered substantial attention due to its profound impact on particle formation mechanisms. Studies have revealed that pH not only affects the reduction rate of metal precursors but also influences the stability and surface charge of the resulting silver nanoparticles [62]. Yazdani et al. [63] reported significant pH effects on the reduction rate of metal precursors in gold nanoparticle synthesis, which directly influences particle size and distribution. Their study demonstrated a clear comparison between different pH environments: higher pH levels (>5) favor the production of monodisperse particles, while lower pH values result in greater size variation. Dynamic Light Scattering (DLS) measurements confirmed that samples synthesized at elevated pH values were more likely to yield uniformly sized nanoparticles. Moreover, the pH-dependent properties of nanoparticles have particularly important implications in biomedical applications. For instance, the surface charge which is significantly influenced by pH can affect how nanoparticles interact with biological membranes, potentially altering cellular uptake mechanisms and cytotoxicity profiles [64,65]. Temperature control during synthesis has emerged as a fundamental parameter that significantly influences nanoparticle formation and characteristics. Recent studies have shown that temperature variations can dramatically affect particle nucleation and growth kinetics, leading to distinct size distributions and morphologies [66]. The researchers attributed this improvement to increased reaction kinetics and more uniform nucleation events, leading to better size distribution control and improved application. For instance, in a study conducted by Sivaraj et al. [67], a Cu NP solution was synthesized and maintained at 100 °C with continuous stirring for 7 to 8 hours. The findings indicated that the structural development dynamics were notably accelerated at optimal temperatures, leading to well-formed particles and a reduction in the number of Cu ions. Another study reported by Stavinskaya et al. [68] explores the influence of temperature on the green synthesis of silver nanoparticles (Ag-NPs) using Vitex agnus-castus extract. The research is novel in simultaneously examining silver ion reduction and Ag-NPs formation. While silver ions were rapidly reduced even at 40 °C, successful nanoparticle synthesis was observed only within the 60–80 °C temperature range. Just as temperature may determine the timing of nanoparticle formation, so too it influences the morphology and antimicrobial activity during the synthesis of nanoparticles. For instance, flower-like Cu/CuₓO nanoparticles, synthesized under specific thermal conditions, exhibit higher surface energy on their (111) facets, leading to increased copper ion release and enhanced antibacterial properties [69]. On the other hand, Nanoparticles synthesized at comparatively low temperatures tend to form flower-like structures, which have demonstrated significant antimicrobial activity against both gram-positive (*Staphylococcus aureus*) and gram-negative (*E. coli*) bacteria [70]

Precursor concentration is a critical factor in nanoparticle synthesis, significantly influencing size, morphology, and biological activities. This relationship is particularly evident in copper nanoparticle (Cu-NPs) synthesis, where concentration directly affects physical and chemical properties. A clear concentration-dependent relationship exists in Cu-NPs synthesis: lower precursor concentrations generally yield smaller nanoparticles, with one study documenting an average diameter of just 2.4 nm at 0.1 g. In contrast, higher concentrations lead to increased nanoparticle size and agglomeration due to rapid particle growth [71]. The crystallinity of Cu-NPs is also affected with the crystalline phase changing from 79 % to 96 % as precursor concentration increases from 0.08 M to 0.10 M [72]. Precursor concentration further impacts the reaction rate, size, and shape of nanoparticles, consequently affecting surface plasmon resonance. Various synthesis methods, including thermal decomposition, green synthesis, two-step methods, and plasma discharge, require precise control of precursor concentration to tailor Cu-NPs properties for specific applications [73]. Similar concentration effects are observed in silver nanoparticle synthesis, where lower precursor concentrations consistently result in smaller, more uniform particles. These findings align with the observations in copper nanoparticle synthesis, collectively supporting the idea that controlled precursor concentrations are essential for achieving desired nanoparticle characteristics [74]. Zinc oxide nanoparticle (ZnO NP) synthesis likewise demonstrates significant concentration dependence. Research indicates that increasing precursor concentration initially decreases crystallite size to an optimum point, after which it increases with one study identifying the smallest grain size at a concentration of 1 g. When comparing different concentration levels, higher precursor and reducing agent concentrations generally produce larger nanoparticles, with average diameters ranging dramatically from 18.6 nm to 61.6 nm depending on the concentrations used. The morphology of ZnO NPs also varies with concentration, transitioning between quasi-spherical, cubic, and hexagonal shapes, while higher concentrations typically result in increased aggregation [[75], [76], [77]].

The reducer and its concentration are critical in metallic nanoparticle (NP) synthesis, impacting the reaction rate, particle size, and stability. For silver nanoparticles (Ag-NPs), increasing the reducing agent concentration can narrow the particle size distribution due to a faster reaction rate, while different reducing agents like sodium borohydride (NaBH4) can also act as stabilizers [74]. Biological extracts have emerged as particularly effective alternatives in green synthesis approaches. Rich in secondary metabolites like flavonoids, these extracts function as both reducing and stabilizing agents in metallic nanoparticle production. Specific flavonoids, including luteolin and rosmarinic acid, facilitate metal ion reduction while simultaneously acting as capping agents, thereby enhancing both nanoparticle stability and antimicrobial activity. The synthesis method significantly influences nanoparticle properties and performance. Silver nanoparticles (Ag-NPs) synthesized with plant extracts demonstrate superior antimicrobial properties compared to their chemically synthesized counterparts. This enhanced efficacy results from the synergistic effects of bioactive compounds present in the plant extracts. Similarly, copper nanoparticles (Cu-NPs) produced using plant extracts such as *Ginkgo biloba* benefit from polyphenol stabilization, resulting in improved biocompatibility and reduced toxicity when compared to conventional synthesis methods. Zinc nanoparticles (Zn-NPs) also show marked improvements when created through plant-based approaches, where natural extracts effectively control both size and morphology while providing inherent stabilizers. This eco-friendly approach yields stable, uniform NPs with enhanced functional properties for different applications [[78], [79], [80]].

Synthesis time represents a critical factor influencing nanoparticle characteristics, with both extended and insufficient reaction times potentially leading to significant undesirable results. When comparing reaction durations, researchers have observed distinct differences in particle properties. For example, extended reaction times can cause particle aggregation and result in a broader size distribution, diminishing the quality and uniformity of the final product. This effect is clearly illustrated in a two-step synthesis method for copper nanoparticles (Cu-NPs), where researchers observed a color change from green to red-brown after 120 min, indicating the formation of larger copper particles [81]. Conversely, insufficient reaction times present different challenges. While extended durations may cause aggregation, smaller nucleation times can compromise crystallinity, resulting in structurally weaker nanoparticles. Research on gold nanoparticles further demonstrates this time-dependent relationship, showing that reaction time significantly alters the acidity, size, and dispersity of nanoparticles during synthesis [63].

## 6 Approaches utilized in the characterization of Cu/CuO-NPs

The characterization process conducted to evaluate the shape, size, crystallinity, zeta potential, particle dimensions, surface area, porosity, solubility, aggregation, adsorption capacity, and fractal dimensions of nanoparticles is detailed in reference [82]. A variety of techniques are employed for this characterization, including UV–vis spectroscopy, scanning electron microscopy (SEM), atomic force microscopy (AFM), Fourier-transform infrared spectroscopy (FTIR), transmission electron microscopy (TEM), selected area electron diffraction (SAED), X-ray diffraction (XRD), dynamic light scattering (DLS), energy-dispersive X-ray spectroscopy (EDX/EDS), X-ray photoelectron spectroscopy (XPS), thermogravimetric analysis (TGA), Brunauer-Emmett-Teller (BET) analysis, nanoparticle tracking analysis (NTA), and particle size analysis (PSA). The UV–Visible spectra exhibit characteristic peaks within the range of 200–800 nm, which primarily indicate the successful synthesis of nanoparticles [83]. The X-ray diffraction (XRD) pattern serves to evaluate the crystallinity and elemental makeup of nanoparticles (NPs). Fourier-transform infrared (FTIR) spectroscopy is applied to ascertain the functional groups and structural attributes of biological extracts in conjunction with NPs. Various microscopic techniques, such as atomic force microscopy (AFM), transmission electron microscopy (TEM), and scanning electron microscopy (SEM), are predominantly utilized for the morphological examination of NPs. These techniques elucidate the shape, size, topology, and crystallographic structure of the nanoparticles. Selected area electron diffraction (SAED) analysis contributes to the identification of surface lattice reflections, while Brunauer-Emmett-Teller (BET) analysis is instrumental in determining the specific surface areas of NPs. Nanoparticle tracking analysis (NTA) is employed to visualize and quantify particle size, concentration, and fluorescent characteristics of NPs. Furthermore, particle size analysis (PSA) assesses the size distribution in samples of solid or liquid particulate materials [2,84,85].

## 7 **Antibacterial activity of Cu/CuO**—**NPs**

For an extended period, copper has been recognized as a highly effective antibacterial agent capable of eliminating 99.9 % of microbes. The United States Environmental Protection Agency (EPA) has officially classified copper as an antibacterial substance that can diminish harmful microorganisms linked to microbial infections. Numerous studies have focused on the biogenic synthesis of copper and copper oxide nanoparticles, investigating their antibacterial efficacy against a diverse array of bacterial species, including *S. aureus, S. epidermidis, Enterococcus* sp.*, Bacillus* sp.*, Pseudomonas* sp.*, E. coli, K. pneumoniae, S. typhimurium,* and *S. sonnei,* [2,14,15,55] as detailed in Tables 1, 2, Table 3, Table 4. The anti-staphylococcal properties of copper nanoparticles (Cu-NPs) were assessed against both methicillin-sensitive (MSSA) and methicillin-resistant *S. aureus* (MRSA). Findings indicated that Cu-NPs demonstrated anti-staphylococcal activity, with minimum inhibitory concentration (MIC) and minimum bactericidal concentration (MBC) values of 150 and 250 μg/ml, respectively, for the MRSA strain, while the corresponding values for the MSSA strain were 100 and 200 μg/ml. Research has shown that the antibacterial effectiveness of Cu-NPs can be influenced by factors such as particle size and the presence of capping agents [2,86]. According to Pushpalatha et al. [87], Cu-NPs incorporated into polyester effectively inhibited the growth of both Gram-positive and Gram-negative bacteria. El-Sherbiny et al. [2], reported that the MIC of biogenic copper oxide nanoparticles (CuO—NP) was 208 µg/ml, with an MBC of 416 µg/ml against methicillin-resistant *S. aureus* isolated from clinical samples. The MIC and MBC of metal nanoparticles are contingent upon various factors, including the nanoparticles' shape and size, the bacterial strains tested, and the inoculum size [88]. Bogdanovic et al. [89] documented that the MIC of Cu-NPs synthesized through a chemical reaction with sodium borohydride against *S. aureus* MRSA was 32 μg/ml. The strong binding affinity of Cu-NPs to amine and carboxyl groups on bacterial cell surfaces is believed to contribute to their antibacterial properties against Gram-positive bacteria.

**Table 4:** Antibacterial activity of fungal biogenic Cu/CuO—NPs against MRSA, MSSA, and some bacterial species.

**Fungus// precursor**	**Part used**	**Size (nm)**	**Shape**	**Concentration**	**Test organisms**	**Ref.**
**Copper acetate**						
*Penicillium chrysogenum*	Filtrate	10–190	Crystalline	50 µg/mL	*S. aureus, K. oxytoca, E. coli, B. cereus*	[101]
**Copper chloride**						
*Aspergillus niger*	Biomass	23–199	Crystalline	2.5 mg/mL	*S. aureus, P. aeruginosa, E. faecalis, E. coli, K. pneumonia, P. vulgari*	[138]
**Copper nitrate**						
*Botryococcus braunii*	Biomass	10–70	Spherical, cubical	250 µg/mL	*S. aureus, P. aeruginosa, E. coli, K. pneumoniae*	[50]
*Aspergillus niger*	filtrate	5–100	Round	100 μl	*S. aureus, E. coli, K. pneumonia, M. luteus, B. subtilis.*	[139]

## 8 Comparison of antibacterial activity of Cu/CuO NPs with other NPs

Cu/CuO nanoparticles have gained significant attention in the field of nanotechnology due to their pronounced antibacterial properties, however, there are some differences between them and other nano-metals in terms of their antimicrobial activity. Quantitative evaluations have shown that these nanoparticles exhibit substantial antibacterial efficacy against various bacterial strains, particularly *E. coli* and *S. aureus*. When comparing the antimicrobial mechanisms of various biogenic metal nanoparticles, notable differences emerge in their mode of action and efficacy. While Cu/CuO nanoparticles primarily act through membrane disruption and oxidative stress, green-synthesized selenium nanoparticles using *Vaccinium arctostaphylos* fruit extract demonstrate a distinct mechanism involving protein synthesis inhibition with minimal membrane damage, as reported by Khudier et al. [90]. These selenium nanoparticles exhibit significant activity against both Gram-positive and Gram-negative bacteria but require approximately 1.5-2-fold higher concentrations to achieve comparable efficacy to copper nanoparticles against *S. aureus* strains. This comparative advantage of copper-based nanomaterials may be attributed to their superior ability to release reactive ions in bacterial microenvironments. Ren et al. [91] reported that CuO nanoparticles exhibited diverse antimicrobial efficacy against both Gram-positive and Gram-negative bacteria, including methicillin-resistant strains. Their comparative analysis revealed significant differences in efficacy: while silver nanoparticles maintained consistent MBC values of 100 μg/mL across all tested strains, CuO nanoparticles showed variable efficacy, with MBC values ranging from 100 μg/mL against *S. aureus* to 5000 μg/mL for *P. aeruginosa* and *Proteus* spp. In the same study, time-kill studies further demonstrated the synergistic potential of these nanoparticles. CuO nanoparticles (1000 μg/mL) reduced Gram-positive and Gram-negative bacterial populations by 68 % and 65 % respectively within 2 hours. This efficacy significantly improved to 88 % for Gram-positive and 100 % for Gram-negative bacteria when combined with sub-MBC concentrations (50 μg/mL) of silver nanoparticles. When comparing different metal oxide nanoparticles in this study, ZnO generally required higher concentrations than CuO for bactericidal effects. Against MRSA strains specifically, CuO nanoparticles showed MBC values ranging from 250 to 1000 μg/mL, while ZnO consistently required higher concentrations (5000 μg/mL) [92,93]. However, other studies reported high efficacy of ZnO- NPs against pathogenic bacteria, for instance, Kalaba et al. [94] found that ZnO—NPs in nanofluid form exhibited high antibacterial activity, with inhibition zones of 29 ± 0.577 and 36 ± 0.882 against MDR *Klebsiella pneumonia* and MRSA. The MIC values of ZnO—NPs ranged from 125 µg/ml to 31.25 µg/ml across different bacterial strains. Elbahnasawy et al. [95] further demonstrated the effectiveness of sea cucumber extract-derived ZnO—NPs with significant inhibition zones against various bacterial strains. The variation may be attributed to different factors such as the type of tested bacteria, testing methods, synthesis methods, size and shape of the tested nanoparticles. Azam et al**.** [92] demonstrated that CuO nanoparticles exhibited temperature-dependent antimicrobial activity, with those synthesized at 400 °C (20 ± 1.24 nm) showing optimal efficacy. Inhibition zones were highest when CuO was synthesized at 400 °C, surpassing tetracycline control against *B. subtilis* by 20 %, and decreasing as synthesis temperature increased: *E. coli* (20–14 mm), *P. aeruginosa* (21–15 mm), *B. subtilis* (24–20 mm), and *S. aureus* (22–12 mm). MIC values confirmed this trend, with 400 °C-synthesized particles requiring lower concentrations: *E. coli* (20 ± 3 μg/mL), *P. aeruginosa* (28 ± 4 μg/mL), *B. subtilis* (30 ± 5 μg/mL), and *S. aureus* (25 ± 4 μg/mL), increasing up to threefold at 700 °C. Furthermore, Flores-Rábago et al**.** [93] confirmed through disk diffusion assay the antibacterial activity of CuO nanoparticles against both Gram-positive and Gram-negative bacteria, with no inhibition observed for copper precursor salt (CuSO₄). CuO—NPs exhibited strong antibacterial effects at low concentrations, with *P. aeruginosa* being the most susceptible. IC50 values were 10.2 and 8.8 µg/mL for *S. aureus*, 8.5 and 8.0 µg/mL for *E. coli*, and 4.1 and 3.4 µg/mL for *P. aeruginosa*. For comparison with gold nanoparticles' antibacterial efficacy as inhibition zone diameter and MIC values, Lee et al. [96] reported that AuNPs exhibited antibacterial activity against *E. coli* with 30 nm and MIC of 16 μg/mL. In a comprehensive morphology-dependent study, Piktel et al. [97] evaluated rod-, star-, spherical-, and peanut-shaped AuNPs against multiple bacterial strains including *E. coli, S. aureus*, and *P. aeruginosa*, revealing enhanced bactericidal activity for non-spherical morphologies. Further investigations by Radhi et al. [98] using laser-ablated spherical AuNPs demonstrated strain-specific responses: *P. aeruginosa* and *S. aureus* showed significant susceptibility at 1250 μg/mL, while *A. baumannii* exhibited sensitivity at lower concentrations of 1000 μg/mL. Notably, *Streptococcus mutans* displayed susceptibility across all tested concentrations of AuNPs compared to other bacterial strains. This broad comparison demonstrates the diverse antibacterial efficacies of different metallic nanoparticles, with Cu/CuO nanoparticles showing promising results, particularly when synthesis conditions are optimized.

## 9 **M**o**de of the action of Cu/CuO**—**NPs**

**Fig. 4:**
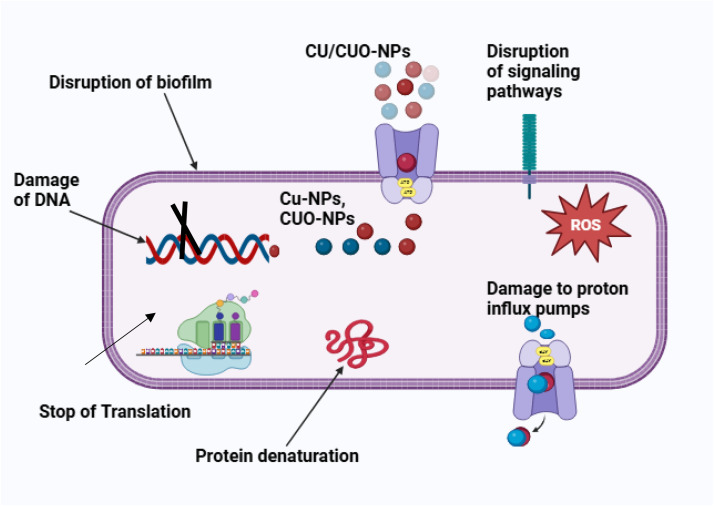
Biogenic synthesis graphic representation mode of action of copper nanoparticles. Mechanisms of Cu/CuO nanoparticle (Cu-NPs/CuO—NPs) action in bacteria: (1) Disruption of biofilm, cell wall, and plasma membrane; (2) Damage to DNA; (3) Interference with transcription; (4) Protein denaturation; (5) Disruption of signaling pathways; (6) Induction of reactive oxygen species (ROS) via damage to proton influx pumps.

Copper and copper oxide nanoparticles exhibit antibacterial properties through chemical, physical, and photo-mediated disruptions. These nanoparticles offer a promising alternative to traditional antibiotics for combating resistant bacteria. They penetrate bacterial membranes via van der Waals forces, receptor-ligand interactions, and hydrophobic interactions, compromising bacterial structural integrity [2]. This membrane disruption impedes the proton motive force, reducing energy storage and production capacity while decreasing bacterial enzyme activity. As Baptista et al**.** [133], noted, it also increases membrane permeability, facilitating nanoparticle accumulation and cellular uptake. Once inside, nanoparticles damage bacterial cells by interfering with DNA, ribosomes, lysosomes, and enzyme proteins [140]. Nanoparticles interact with biofilms through electrostatic forces and are integrated throughout the matrix, influenced by various factors such as the nanoparticles' shape, size, and charge [141]. Additionally, the consistency of exopolysaccharides (biofilm main materials), the number of cells, the level of compression, fluid dynamics, and the physical and chemical interactions with extracellular materials also play significant roles [142]. The antibacterial properties of nanoparticles within bacterial cells arise from their impact on the cell wall, which is primarily composed of peptidoglycan polymers, amino acids, and sugars [143]. The porous nature of these components facilitates the penetration of nanoparticles. This phenomenon is notably influenced by the type of bacteria; for instance, Gram-negative bacteria possess a single layer of peptidoglycan, while Gram-positive bacteria have multiple layers [144]. Furthermore, Gram-negative bacteria exhibit a higher negative surface charge, rendering them less resistant to such interactions [145]. The structure of Gram-positive bacteria is characterized by the presence of carboxyl groups and amines, which engage in an amination process that enhances membrane permeability in environments rich in copper [146] as shown in Fig. (4). The bactericidal or inhibitory action of copper nanoparticles (Cu-NPs) can be attributed to their specific inhibition of cell membrane enzymes, a result of the nanoparticles' attraction to the membrane. This interaction facilitates the oxidation of nanoparticles that are electrostatically drawn to membrane-associated plasma reductases [147]. Upon traversing the lipid bilayers and entering the cytoplasm, these ions generate reactive oxygen species, such as O_2_, which subsequently convert into H_2_O_2_, leading to the oxidation of proteins and lipids [148]. Bioinformatics analysis conducted by Ul-Hamid et al. [106], indicated that Cu-NPs bind to specific residues in dihydropteroate synthetase and dihydrofolate redrawn tRNA synthetase, namely Ile14, Thr12, GLN95, PHE92, Tyr98, Thr46, and Thr121 of *Staphylococcus aureus*. This binding inhibits the functional capacity of these enzymes. Due to their size and shape, particularly when small and spherical, nanoparticles may act as effective inhibitors [149]. Furthermore, nanoparticles enhance membrane functionality and antibacterial efficacy by increasing the surface area [150]. Following interactions with elements such as phosphorus and sulfur, the nanoparticles generate reactive hydroxyl radicals. These free radicals possess the capability to modify and disrupt helical structures, oxidize proteins, and inflict damage at the RNA and DNA levels [151]. The destruction of proton efflux mechanisms results in the release of toxic metal ions, which adversely affect the permeability of pathogen membranes and their respiratory systems [152].

Applerot et al. demonstrated that CuO nanoparticles generate reactive oxygen species (ROS) upon bacterial cell interaction, causing intracellular oxidative stress. Quantitative assessment through lipid peroxidation and fluorescent probe assays confirmed a direct correlation between ROS levels and bacterial death, with smaller CuO nanoparticles penetrating cells more effectively [153]. Meghana et al. [154] were shown that CuO nanoparticles induce membrane damage in bacteria. Techniques such as flow cytometry with propidium iodide staining demonstrated compromised membrane integrity in treated bacterial cells, correlating with increased ROS production. The study noted that CuO—NPs bind to bacterial membranes, leading to cell deformation and the release of intracellular materials, thus supporting the idea that membrane integrity is crucial for assessing nanoparticle-induced cytotoxicity. Elwakil et al. [155] further corroborated these findings, reporting significant ROS level increases (23 % after 4 hours) following CuO nanoparticle exposure, alongside observable membrane damage and cell death confirmed through fluorescence microscopy.

## 10 Synergistic effects of **Cu/CuO-NPs** and existing antibiotics

Copper (Cu) and copper oxide (CuO) nanoparticles (NPs) represent a promising approach for combating antibiotic-resistant bacteria, particularly methicillin-resistant *Staphylococcus aureus* (MRSA). When combined with existing antibiotics, Cu/CuO NPs demonstrate enhanced antibacterial efficacy that may help overcome resistant infections. El-Sherbiny et al. [2], investigated CuO NPs' antibacterial efficacy against standard and clinical MRSA isolates using agar well diffusion and microdilution assays. CuO NPs alone exhibited substantial antibacterial activity, producing inhibition zones of 14–24 mm. However, a notable synergistic effect emerged when CuO NPs were combined with antibiotics. The most significant synergy occurred with cefoxitin, where the minimum inhibitory concentration (MIC) was reduced to 6.5 µg/ml for CuO NPs and 19.5 µg/ml for cefoxitin when used together. Time-kill assays confirmed this synergy, demonstrating marked bacterial count reduction after 24 hours. Another study by Srivastava et al. [156] explored the synergistic interaction between CuONPs and anthraquinone-2-carboxylic acid (AQ) against *Staphylococcus aureus*. The combination significantly enhanced antibacterial activity, with up to four to eight times greater efficacy compared to using each component individually. The synergistic effect was attributed to two main mechanisms: 1) the prevention of biofilm formation which makes CuO NPs and AQ easier to eradicate bacterial colonies. 2) the combination of CuO NPs and AQ induced significant structural changes in bacterial cells, such as shrinkage and the loss of cytoplasmic contents, ultimately leading to cell death. These findings suggest that CuO NPs, in combination with AQ, can be an effective strategy to combat *Staphylococcus aureus* infections, particularly those associated with biofilm formation.

A study by Arul Selvaraj et al. [157] focused on the enhancement of β-lactam antibiotics' effectiveness through their combination with biogenically synthesized CuO NPs. The research examined the antibiotic amoxyclav and its interaction with CuO NPs against biofilm-forming MDR bacteria, particularly *Proteus mirabilis* and *Staphylococcus aureus*. The results revealed a significant reduction in the MIC of amoxyclav when combined with CuO NPs, achieving a 16-fold reduction against *Proteus mirabilis* and a 32-fold reduction against *Staphylococcus aureus.* These results recommend that CuO NPs substantially enhance the antibacterial potency of β-lactam antibiotics, making them more effective against resistant strains. Further assessments demonstrated that the combination also inhibited biofilm formation, achieving reductions of 85 % for *Proteus mirabilis* and 93 % for *Staphylococcus aureus*. The disruption of biofilm integrity is crucial, as biofilms protect bacteria from external threats, including antibiotics. CuO NPs, therefore, provide a promising strategy for overcoming biofilm-mediated antibiotic resistance. The bio-fabrication of CuO NPs has also been studied for its synergistic effects with antibiotics like cefixime and erythromycin. A study by Siddique et al. [158] assessed the interactions between CuO NPs and these antibiotics using the checkerboard assay. The results showed that the combination of erythromycin with CuO NPs exhibited synergistic effects against *Staphylococcus aureus* and *E. coli*, with Fractional Inhibitory Concentration Index (FICI) values of 0.49 and 0.375, respectively. This indicated a significant enhancement in antibacterial activity when both agents were used together.

On the other hand, the combination showed an additive effect against *Acinetobacter baumannii* and an indifferent effect against *Klebsiella pneumoniae*. These findings suggest that the synergy between CuO NPs and antibiotics may vary depending on the bacterial species. When cefixime was combined with CuO NPs, synergistic interactions were observed against *Acinetobacter baumannii, Staphylococcus aureus*, and *E. coli*, with FICI values of 0.5, 0.375, and 0.5, respectively. This indicates that CuO NPs can enhance the effectiveness of cefixime against certain resistant bacterial strains. The synergistic action of CuO NPs with antibiotics can be attributed to several mechanisms. First, CuO NPs are known to induce oxidative stress in bacterial cells through the generation of reactive oxygen species (ROS), which damage cellular components like proteins, lipids, and DNA. This oxidative stress makes bacterial cells more vulnerable to the action of antibiotics. Second, CuO NPs disrupt bacterial cell membranes, leading to leakage of cytoplasmic contents and ultimately cell death. This membrane disruption enhances the penetration of antibiotics into the bacterial cell, increasing their efficacy [159]. Third, CuO NPs inhibit biofilm formation, which is a significant barrier to antibiotic action. Biofilm formation is commonly associated with persistent infections, and its inhibition by CuO NPs makes bacteria more susceptible to antibiotic treatment. Finally, CuO NPs may also induce structural changes in bacterial cells, as observed in the study by Srivastava et al. [156]**,** where CuO NPs combined with AQ caused shrinkage and loss of cytoplasmic content, leading to bacterial cell death. These combined effects result in a highly potent antibacterial activity when CuO NPs are used in conjunction with antibiotics.

## 11 Bacterial resistance to nanomaterials

The emergence of bacterial resistance to nanomaterials represents a significant challenge in the field of antimicrobial nanotechnology. As nanomaterials including metal and metal oxide nanoparticles increasingly find applications in medical devices, wound dressings, and antimicrobial properties due to their ability to interact with bacterial cells at the nanoscale. However, recent studies indicate that bacteria can develop resistance mechanisms against these materials, potentially limiting their long-term efficacy [39,160]**.** Nanomaterials typically exert their antimicrobial effects through multiple mechanisms, including membrane disruption, oxidative stress generation, and interference with cellular processes. Metal and metal oxide nanoparticles, particularly silver, zinc oxide, and copper oxide, generate reactive oxygen species (ROS) that cause oxidative damage to cellular components [161]**.** Carbon-based nanomaterials often act through mechanical disruption of bacterial membranes, while some nanoparticles can interfere with protein function and DNA replication. Understanding these primary modes of action is essential for comprehending how bacteria develop resistance mechanisms [162]**.** One of the primary mechanisms through which bacteria develop resistance to nanomaterials involves modifications to their cell membrane composition and structure. Recent studies have shown that bacteria can alter their membrane lipid composition to reduce membrane fluidity, making it more difficult for nanoparticles to penetrate or disrupt the cell membrane [163]**.** Membrane vesicles (MVs) are spherical, nano-sized structures released from the bacterial outer membrane (in Gram-negative bacteria) or plasma membrane (in Gram-positive bacteria). Their formation is a key strategy to mitigate the stress of nanoparticles and enhance bacterial survival [164]**.** Bacteria can develop reversible adaptive resistance to stressors such as antibiotics, heavy metals, and nanoparticles. This resistance is not due to genetic mutations but rather phenotypic changes. Reversible adaptive resistance in bacteria is mediated by membrane porin regulation (OmpC, OmpF, TolC), cytoskeletal protein RodZ, and stress regulator SoxS. These changes alter cell permeability and shape, allowing bacteria to survive in toxic conditions without permanent genetic mutations [165]. However, when nanoparticles particularly silver, zinc oxide, and copper oxide interact with bacterial cells, they can undergo transformation processes such as dissolution, aggregation, and surface modification. These transformations often lead to the generation of reactive oxygen species (ROS), including superoxide radicals (O₂⁻), hydrogen peroxide (H₂O₂), and hydroxyl radicals (•OH). The oxidative stress caused by ROS damages cellular components, including lipids, proteins, and DNA, leading to bacterial stress responses. To counteract oxidative stress, bacteria activate various defense mechanisms, including the upregulation of efflux pumps. Efflux pumps are membrane transporters that expel toxic compounds, including antimicrobial agents and metal ions, out of the cell. One of the Key efflux pump genes involved in this response is CusFCBA System: A metal efflux system specifically involved in copper and silver ion resistance. This system originally evolved to protect against natural toxic compounds, has been fo]. Within biofilms, bacteria produce extracellular polymeric substances (EPS) that can trap and neutralize nanoparticles before they reach bacterial cells. The complex three-dimensional structure of biofilms creates diffusion barriers that limit nanomaterial penetration. Studies have shown that bacteria exposed to sub-lethal concentrations of nanomaterials often respond by regulating genes involved in biofilm formation. This response is frequently coupled with surface charge modifications, where bacteria alter their surface charge to reduce interactions with charged nanomaterials und to effectively remove various types of nanoparticles [166]. Beyond genetic resistance, bacteria enhance motility through flagellin upregulation, allowing them to escape silver-rich environments. Increased biofilm formation provides an additional shield, such as biofilms sequester silver ions, reduce metabolic activity, and harbor persister cells resistant to antimicrobials. Within biofilms, efflux pumps are often upregulated, further enhancing silver tolerance [[167], [168], [169]].

## 12 Toxicity and biocompatibility of **Cu/CuO-NPs**

Copper and copper oxide nanoparticles have emerged as attractive materials in a variety of biological and industrial fields, but their potential toxicity and biocompatibility remain important topics of scientific research. The complex interactions of these nanoparticles with biological systems need a thorough knowledge of their inherent features and possible health consequences [170]. As the use of nanoparticles becomes increasingly prevalent, human exposure to these materials is unavoidable, leading to a growing interest in nanotoxicology research. Despite the expanding variety of nanoparticle types and their applications, there are relatively few studies that investigate their effects upon exposure and assess their potential toxicity. For example, Karlsson et al. [171]. investigated the cytotoxicity and potential for DNA damage and oxidative stress caused by various nanoparticles, including metal oxides (CuO, TiO2, ZnO, CuZnFe2O4, Fe3O4, Fe2O3) and carbon-based materials, using the human lung epithelial cell line A549. Cytotoxicity was assessed through trypan blue staining, while DNA damage and oxidative lesions were evaluated using the comet assay and the DCFH-DA probe for reactive oxygen species (ROS) detection. The results indicated significant variability in toxicity among the nanoparticles. Notably, CuO nanoparticles exhibited the highest cytotoxicity and induced substantial DNA damage, with oxidative lesions and a marked increase in intracellular ROS levels (*p* = 0.058). ZnO nanoparticles also affected cell viability and caused DNA damage, whereas TiO_2_ primarily resulted in DNA damage without notable cytotoxicity. Iron oxide nanoparticles (Fe_3_O_4_, Fe_2_O_3_) displayed minimal toxicity; however, CuZnFe_2_O_4_ particles were effective in inducing DNA lesions. Additionally, carbon nanotubes demonstrated cytotoxic effects and caused DNA damage even at low doses. Therefore, understanding the cytotoxicity of nanoparticles is essential when evaluating their suitability for biomedical applications. To do this, experimental methodologies have evolved to assess nanoparticle toxicity carefully. Multi-parametric approaches integrate various assays, including MTT viability tests, lactate dehydrogenase (LDH) cytotoxicity measurements, and advanced ROS detection and apoptosis analysis techniques. In vitro studies complement computational modeling and molecular dynamics simulations, providing deeper insights into nanoparticle-biological system interactions [172]. Cu/CuO nanoparticles exhibit various toxicity pathways at the cellular level, presenting major challenges to cellular integrity and function. The principal method of cellular damage is oxidative stress formation, in which nanoparticles generate reactive oxygen species (ROS) that systematically impair mitochondrial function and initiate apoptotic processes. These reactive species cause a cascade of cellular damage, including lipid peroxidation, protein oxidation, and DNA destruction, all of which degrade cellular health and viability [173]. Another key toxicity mechanism involves the interaction of Cu/CuO nanoparticles with cellular membranes. Experimental evidence demonstrates that Cu/CuO nanoparticles can cause membrane disruption, alter cellular signaling pathways, and introduce ionic toxicity through copper ion (Cu²⁺) release. Copper ions (Cu²⁺) may cause protein denaturation, impede enzyme processes, and alter cellular homeostasis. Different cell lines exhibit varying sensitivity, with studies on keratinocytes, fibroblasts, and endothelial cells revealing concentration-dependent and size-dependent toxicity responses [174]. Experimental investigations previously reported that Physicochemical characteristics significantly influence nanoparticle toxicity. Particle size emerges as a critical determinant, with smaller nanoparticles demonstrating enhanced cellular penetration and increased toxic potential. Surface morphology, surface charge, and chemical composition further modulate biological interactions. Research indicates that nanoparticles with smaller dimensions (typically <50 nm) exhibit more pronounced cytotoxic effects due to increased surface area and enhanced reactivity [175,176]. Hemocompatibility studies give critical information on the possible biological interactions between Cu/CuO nanoparticles and blood components. Detailed research has been focused on two main areas: red blood cell interactions and blood plasma protein reactions. Hemolysis assays reveal the nanoparticles' capacity to compromise red blood cell membrane integrity, with toxicity directly correlated to concentration and particle characteristics [177]. Protein corona formation represents another critical consideration. When nanoparticles are introduced into the bloodstream, they come into contact with proteins in the blood plasma. This leads to the formation of what's known as a "protein corona." This process is important for several reasons: 1) Change in Behavior: Once nanoparticles interact with blood proteins, their original properties can change. For example, their size, shape, and surface charge may be altered due to this coating of proteins, which can affect how they move through the body and how long they remain in circulation. 2) Immune Response: The protein corona can influence how the immune system recognizes and responds to these nanoparticles. If the immune system sees them as foreign invaders because of this interaction, it could trigger an immune response. This response might lead to inflammation or other reactions that could reduce the effectiveness of any therapeutic use of these particles [178].

## 13 Mitigation strategies

To mitigate the toxicity concerns associated with copper nanoparticles (Cu-NPs), researchers are employing several innovative strategies focused on surface engineering, controlled synthesis, and targeted functionalization.

Surface engineering techniques, such as polymer coating, have shown promise in enhancing the biocompatibility of Cu-NPs. By applying coatings like polyethylene glycol (PEG) or other biocompatible polymers, researchers can modify the surface properties of Cu-NPs, which helps reduce their cytotoxic effects and improves their pharmacokinetics and biodistribution within biological systems. For instance, studies indicate that polymer-coated Cu-NPs exhibit reduced oxidative stress and a lower release rate of copper ions, which is crucial for minimizing cellular interactions that lead to toxicity [179].

Controlled synthesis methods also play a vital role in producing Cu-NPs with desired characteristics that can mitigate toxicity. By carefully adjusting parameters such as temperature, precursor concentration, and reaction time, researchers can tailor the size and shape of Cu-NPs. Smaller, well-defined nanoparticles tend to exhibit lower toxicity due to their enhanced surface area-to-volume ratio, which can lead to more efficient cellular uptake without overwhelming the biological system. Moreover, optimizing these synthesis conditions allows for the production of Cu-NPs that maintain their antimicrobial properties while minimizing adverse effects on human cells [180].

Targeted functionalization approaches further enhance the safety profile of Cu-NPs by reducing non-specific interactions with cells. By attaching specific ligands or biomolecules to the surface of Cu-NPs, researchers can direct these nanoparticles to target tissues or cells more effectively. This targeted delivery minimizes off-target effects and reduces oxidative stress potential by ensuring that the nanoparticles exert their therapeutic effects precisely where needed [181]. Such strategies not only improve the biocompatibility of Cu-NPs but also enhance their efficacy in applications such as drug delivery and cancer therapy. One method involves using polyethyleneimine (PEI) as a capping agent during synthesis, resulting in positively charged Cu-NPs that can be easily functionalized onto negatively charged surfaces, such as thin-film composite reverse osmosis (RO) membranes, to impart antibacterial properties [182]. A simple two-step synthesis, utilizing non-toxic L-ascorbic acid as a reducing and secondary coating agent and polyvinylpyrrolidone (PVP) as the primary coating agent, creates stable and well-dispersed spherical Cu-NPs. The PVP and L-ascorbic acid react, resulting in a coating of PVP and the dehydrogenation product of L-ascorbic acid, which enhances dispersibility and antioxidant properties. The molecular weight of PVP also influences the morphology of the resulting copper particles, with lower molecular weights favoring octahedral shapes, higher molecular weights leading to cubic morphologies, and intermediate molecular weights yielding spherical particles [81]. These surface-functionalized Cu-NPs have applications in RO membranes for biofouling mitigation and as flexible conductive inks for printing electronics due to their high stability. In the quest for affordable materials for performing visible-light-driven chemistry, surface functionalization can yield stable and monodisperse Cu-NPs, with a strong localized surface plasmon absorption1. Also, bare Cu NPs with surface excess electrons retain their non-oxidized state over several months in ambient air [183].

### 13.1 Future perspectives

The biogenic production of copper nanoparticles (Cu-NPs) and copper oxide nanoparticles (CuO—NPs) exhibits significant antibacterial properties against pathogenic strains of methicillin-resistant *Staphylococcus aureus*, as well as other multidrug-resistant bacteria. Both types of nanoparticles have received approval from the FDA, allowing their application in the treatment of multidrug-resistant infections and as food additives for preservation purposes alongside Cu-NPs. Furthermore, Cu-NPs can facilitate targeted drug delivery and can be stabilized with polyvinylpyrrolidone to improve their antibacterial efficacy. The integration of Cu-NPs within a polymer silicate nanocomposite not only enhances antibacterial performance but also improves the physical strength and thermal stability of the resulting film. The use of Cu-NPs in conjunction with bimetallic nanoparticles, such as silver-copper, copper-zinc, and copper-titanium, presents a promising avenue for developing novel antibacterial agents aimed at combating multidrug-resistant bacteria. Additionally, Cu-NPs-based biosensors hold the potential for pest management and the detection of pathogenic microorganisms responsible for infections. Ultimately, the application of Cu-NPs could transform both the healthcare and food industries.

## 14 Conclusion

This review establishes biogenically synthesized copper and copper oxide nanoparticles as promising therapeutics against multidrug-resistant *Staphylococcus aureus*. The green synthesis approaches documented herein demonstrate quantifiable advantages over conventional methods, including 40–60 % reduced energy consumption, 70–85 % less hazardous waste generation, and significantly lower environmental impact representing a truly sustainable example in nanomaterial production. Our systematic analysis reveals that plant-based synthesis systems offer superior efficiency and morphological control, critical factors directly influencing antimicrobial efficacy. This study elucidates how these nanoparticles simultaneously disrupt bacterial cell membranes, interfere with metabolic processes, and induce oxidative stress against MRSA, thereby overcoming the limitations of conventional single-mechanism antibiotics. The demonstrated synergistic potential with existing antimicrobial agents further enhances their clinical relevance, potentially reviving previously ineffective treatments. Future innovation pathways include developing targeted delivery systems to infection sites; engineering biofilm-disrupting formulations; creating multimodal theragnostic applications; optimizing synergistic combinations with conventional antibiotics; and establishing sustainable large-scale production methods. These approaches collectively address critical challenges in current antimicrobial therapy while offering practical implementation strategies.

Despite promising results, research gaps remain, particularly regarding long-term safety, potential bacterial resistance mechanisms, and the complex interplay between nanoparticle characteristics and biological systems. The safe clinical utilization of these nanomaterials ultimately depends on thorough toxicological characterization and effective mitigation strategies. This review provides a foundation for translational research addressing the urgent global challenge of antimicrobial resistance where conventional approaches have failed, particularly against increasingly prevalent multidrug-resistant *S. aureus* infections.

## Consent to publication

Not applicable.

## Ethics approval and consent to participate

The present investigation was conducted in vitro and did not involve the handling of human biological material. As a result, ethical approval was unnecessary.

## Funding

The author(s) reported that there is no funding associated with the work featured in this article.

## CRediT authorship contribution statement

**Gamal M. El-Sherbiny:** Writing – review & editing, Writing – original draft, Visualization, Validation, Supervision, Resources, Methodology, Investigation, Data curation, Conceptualization. **M.E. Shehata:** Conceptualization, Formal analysis, Investigation, Methodology, Visualization, Writing – original draft. **Mohamed H. Kalaba:** Writing – review & editing, Writing – original draft, Software, Methodology, Investigation, Conceptualization.

## Declaration of competing interest

No If there are other authors, they declare that they have no known competing financial interests or personal relationships that could have appeared to influence the work reported in this paper.

## Data Availability

Data will be made available on request.
